# Optical coherence tomographic angiography in children with anisometropic amblyopia

**DOI:** 10.1186/s12886-022-02486-9

**Published:** 2022-06-20

**Authors:** Chenchen Liu, Yanzhen Zhang, Xiaopeng Gu, Puying Wei, Dehai Zhu

**Affiliations:** Department of Pediatric Ophthalmology, Peking University First Hospital Peking University Peadatriac Visions Research Center, No.8 Xishiku Street, Xicheng District, Beijing, 100034 China

**Keywords:** Anisometropic amblyopia, Optical coherence tomography angiography, Foveal avascular zone, Vessel density

## Abstract

**Objectives:**

To observe and compare the difference in retinal perfusion using optical coherence tomography angiography (OCTA) between anisometropic amblyopia in children and fellow eyes as well as age-matched controls.

**Methods:**

A total of 16 children with anisometropic amblyopia and 19 age-matched healthy subjects were enrolled. All participants underwent OCTA examination, with 3 mm × 3 mm and 6 mm × 6 mm scans in the macular region. Perfusion parameters of the superficial retinal layer were measured by built-in software, including the macular foveal avascular zone (FAZ) area, perimeter and circularity, as well as the vessel length density (VLD) and perfusion density (PD) of the foveal, parafoveal and perifoveal regions.

**Results:**

Among the 16 patients with anisometropic amblyopia, the FAZ area was significantly higher in diseased eyes (*P *= 0.027) than in fellow eyes. The VLD and PD of the foveal average and the VLD of the nasal quadrant of the perifoveal region in anisometropic amblyopic eyes were significantly lower than those in fellow eyes (*P* < 0.05). The VLD of the parafoveal average, the superior, temporal, inferior and nasal quadrants of the parafoveal region, and the nasal quadrant of the perifoveal region in anisometropic amblyopic eyes were significantly lower than those in healthy controls (*P* < 0.05). The PD of the parafoveal average, and the inferior quadrant of the parafoveal region in anisometropic amblyopic eyes were significantly lower than those in healthy controls (*P* < 0.05). The VLD of the parafoveal average, the superior, inferior and nasal quadrants of the parafoveal region, and the nasal quadrant of the perifoveal region in fellow eyes were significantly lower than those in healthy controls (*P* < 0.05). The PD of the parafoveal average, and the inferior quadrant of the parafoveal region in fellow eyes were significantly lower than those in healthy controls (*P* < 0.05).

**Conclusions:**

The macular vessel density of the superficial capillary plexus is lower in anisometropic amblyopic children than in age-matched healthy children. Compared with the fellow eye, the perfusion of the amblyopic eye in children with anisometropic amblyopia also decreases.

## Background

Amblyopia is a developmental disease of the visual system. Although there was no obvious organic disease in the eye examination, the best corrected visual acuity of one or both eyes did not develop to a normal level. It has been reported that the incidence of the disease in the general population is approximately 2%-5% [1]. Hyperopic anisometropia is one of the causes of amblyopia [2–4]. Amblyopia often occurs in the first 2–3 years of children's growth and development. Amblyopia is generally considered to occur during the development of neurons in the retina and cerebral cortex. It has been reported that the lateral geniculate body and visual cortex are the main affected structures in amblyopia [5–7]. The retina is an important tissue for visual function. The superficial capillaries provide oxygen and nutrients to the superficial retina. However, the relationship between retinal blood flow and amblyopia has not yet been elucidated. OCTA is a novel noninvasive vascular imaging technology that can perform three-dimensional imaging of retinal blood flow, clearly displaying the vascular shape, and it can perform layered quantitative analysis of retinal blood flow. It has been used in the diagnosis and follow-up of a variety of fundus diseases and to investigate the pathogenesis. there are some reports about the use of. Recently, some studies using OCTA to study the macular density and FAZ in children with amblyopia found that the macular density of capillaries increases, but the results remain inconsistent, and there have been few reports on the Chinese population [8–15]. Therefore, the current study aims to use OCTA technology to quantify and compare the blood flow density of superficial retinal capillaries in children with anisometropic amblyopia, in comparison with the fellow eye and age-matched healthy children’s macular area.

## Subjects and methods

### Patients recruitment

This study is a cross-sectional, case–control, noninterventional study. We recruited 16 children with anisometropic amblyopia and 19 age-matched healthy children who were regularly followed up in the Department of Pediatric Ophthalmology, Peking University First Hospital from November 2020 to May 2021. Inclusion criteria were as follows: (1) age 6–15 years old; (2) best corrected visual acuity (BCVA) ≤ 20/30 or BCVA difference of both eyes ≥ 2 line visual standard monocular amblyopic children; (3) at the first visit for patients with anisometropic amblyopia, the diopter (equivalent spherical lens) difference of both eyes is greater than 2.0 diopters (D). The exclusion criteria were as follows: (1) patients with amblyopia or nystagmus deprivation; (2) history of glaucoma, cataracts, retinopathy, optic neuropathy and other ophthalmic diseases; (3) history of eye surgery; and (4) history of preterm birth, neurological diseases or systemic diseases known to affect the retina and choroidal blood vessels such as diabetes.

### Ophthalmological examinations

All subjects underwent ophthalmological examinations, including slit lamp and fundus examination, intraocular pressure, axial length (IOL Master500; Carl Zeiss Meditec, Dublin, CA), cycloplegia refraction and BCVA. Snellen BCVA was converted to logMAR for further analysis. All subjects were examined by the same skilled examiner (Cirrus HD-OCT 5000, Carl Zeiss, USA). The image quality is checked by two readers. The signal intensity is evaluated quantitatively from 1 (poor) to 10 (good), which is automatically evaluated by the machine's built-in software. When the signal strength is less than 7, and artifacts appear due to poor cooperation, such as the patient's blinking, the image quality is judged to be unqualified. Angiography scan mode was selected for the macular area scan and blood flow density parameter measurement, with the macular area as the center, scan the macular area 3 mm × 3 mm and 6 mm × 6 mm, respectively, and the scan depth is 2 mm. The software automatically stratifies the retinal tissue. The built-in AngioPlex software (version 10.0) in the system was employed to identify and measure the blood flow density parameters of the superficial retina in the macular area. The blood flow density in the FAZ, and foveal, parafoveal and perifoveal regions was analysed and measured repeatedly for three times, and the final results were averaged.

### Statistical analysis

The data are first tested for normality and homogeneity of variance. The measurement data with a normal distribution are expressed as the mean ± SD, the. The comparison between the amblyopic eye group and the fellow eye group used the paired sample t test, and the comparison between the amblyopic eye group, the fellow eye group and the normal control group used the independent sample t test. The chi-square test was used to compare the two categorical variables between the two groups. One-way Covariance analysis was used to adjust the axial length and refractive power. *P* < 0.05 indicates that the difference is statistically significant. SPSS statistical software (IBM SPSS, USA, version 23.0) was used for statistical analysis and processing.

## Results

### General data of the patients

A total of 35 subjects with 51 eyes were included in the study, including 16 amblyopic eyes, 16 fellow eyes, and 19 eyes in the healthy control group. Among the patients with amblyopia, 10 were males and 6 were females, with an age of 8.19 ± 2.37 years at diagnosis. The clinical characteristics of the subjects are shown in Tables 1 and 2. Compared with the fellow eye group and the normal control group, the amblyopic eye group had a significantly shorter axial length, higher refractive power and lower LogMAR visual acuity (*P* < 0.05). Compared with the normal control eye group, the fellow eye group had significantly shorter axial length, higher refractive power and lower LogMAR visual acuity (*P* < 0.05).

**Table 1:** Clinical characteristics of the subjects

	Amblyopia eyes (*N* = 16)	Fellow eyes **(***N* = 16)	Healthy control eyes (*N* = 19)
	mean ± SD	mean ± SD	mean ± SD
**Age(years)**	8.19 ± 2.37	8.19 ± 2.37	8.37 ± 2.39
**SEX**			
**MALE**	10(62.5%)	10(62.5%)	7(36.84%)
**FEMALE**	6(37.5%)	6(37.5%)	12(63.16%)
**IOP (MMHG)**	16.44 ± 1.67	16.25 ± 1.65	17.68 ± 2.19
**Axial length(mm)**	21.10 ± 0.73	22.58 ± 0.93	23.28 ± 0.86
**Diopter (D)**	+ 5.27 ± 1.76	+ 1.31 ± 1.61	+ 0.25 ± 0.72
LogMAR	0.44 ± 0.32	0.03 ± 0.04	0

Sex composition is expressed as a percentage

*IOP* intraocular pressure, *LogMAR* logarithm of the minimum angle of resolution, *SD* standard deviation

**Table 2:** Comparison of the Clinical characteristics

	Amblyopia eye with contralateral eye	Contralateral eye with control eye	Amblyopia eye with control eye
	*P* value	*P* value	*P* value
Age	/	0.824	0.824
Sex	/	0.13	0.13
IOP Axial length Diopter LogMAR	0.456 0.000* 0.000* 0.000*	0.439 0.029* 0.024* 0.041*	0.071 0.000* 0.000* 0.000*

*IOP* intraocular pressure, *LogMAR* logarithm of the minimum angle of resolution

^*^P < 0.05, chi-square test was used for sex comparison; the comparison between the amblyopic eye group and the contralateral eye group used the paired sample t test, and the comparison between the amblyopic eye group, the contralateral eye group and the normal control group used the independent sample t test

### FAZ area, perimeter and circularity

**Fig. 1:**
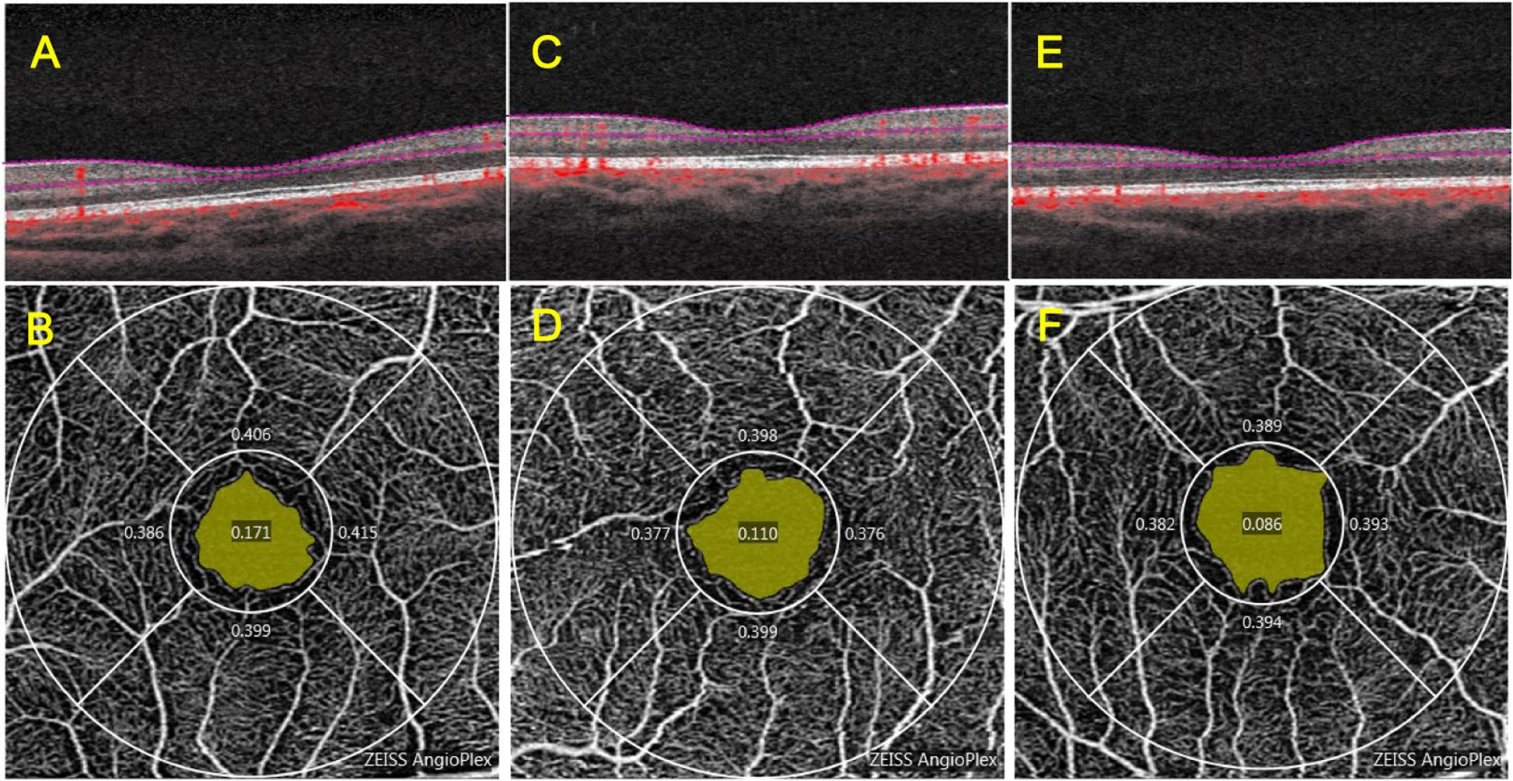
Representative OCTA representative image of the FAZ area, perimeter and circularity measurement. The yellow area in the figure is the FAZ area. A, C and E are horizontal B-scan images passing through the central fovea in the 3 mm × 3 mm region. **A** and **B** are amblyopic eyes, **C** and **D** are contralateral eyes, and **E** and **F** are control eyes. OCTA, optical coherence tomography angiography

The mean superficial FAZ measurements (in order of amblyopic eyes, fellow eyes, control eyes, mm^2^) were 0.329 ± 0.163, 0.298 ± 0.132, and 0.278 ± 0.106. The area of the FAZ in the amblyopic eye group was significantly larger than that in the fellow eye group (*P* = 0.027). There was no significant difference in FAZ perimeter or circularity in the amblyopic eye group compared with the fellow eye and the control eye. There was no significant difference in the FAZ area, perimeter or circularity of the fellow eye compared with the control eye (*P* > 0.05) (Tables 3 and 4). Representative OCTA images are shown in Fig. 1.

**Table 3:** FAZ parameters of the subjects

FAZ parameters	Amblyopia eye *N* = 16	Fellow eye *N* = 16	Control eye *N* = 19
	mean ± SD	mean ± SD	mean ± SD
Area(mm^2^)	0.329 ± 0.163	0.298 ± 0.132	0.278 ± 0.106
Perimeter(mm)	2.332 ± 0.845	2.270 ± 0.560	2.191 ± 0.413
Circularity	0.635 ± 0.196	0.693 ± 0.063	0.690 ± 0.084

*SD* standard deviation

**Table 4:** Comparison of the FAZ parameters

FAZ parameters	Amblyopia eye with contralateral eye	Contralateral eye with control eye	Amblyopia eye with control eye
	*P* value	*P* value	*P* value
Area	0.027*	0.332	0.305
Perimeter	0.563	0.605	0.559
Circularity	0.358	0.171	0.315

^*^P < 0.05, the comparison between the amblyopic eye group and the contralateral eye group used the paired sample t test, and the comparison between the amblyopic eye group, the contralateral eye group and the normal control group used the independent sample t test

### Vessel length density (VLD)

**Fig. 2:**
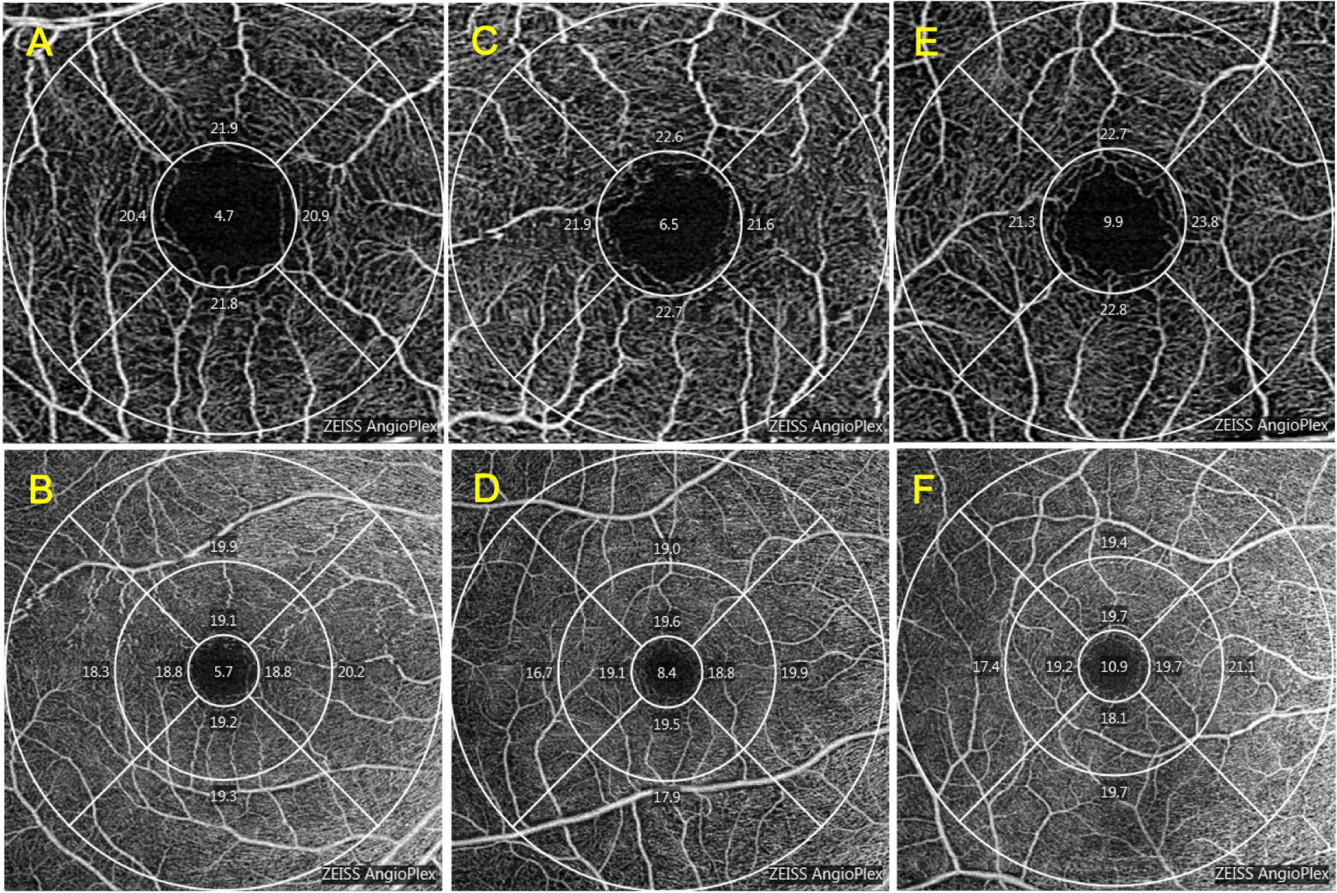
OCTA representative image of VLD. **A** and **B** are amblyopic eyes, **C** and **D** are contralateral eyes, and **E** and **F** are control eyes. The scanning range of **A**, **C** and **E** was 3 mm × 3 mm in the macular area. The scanning range of **B**, **D** and **F** was 6 mm × 6 mm in the macular area. OCTA, optical coherence tomography angiography. VLD, vessel length density

The VLD of the foveal average, and the nasal quadrant of the perifoveal region in anisometropic amblyopic eyes were significantly lower than those in fellow eyes (*P *< 0.05). The VLD of the parafoveal average and the superior, temporal, inferior, nasal quadrants of the parafoveal region and the nasal quadrant of the perifoveal region in anisometropic amblyopic eyes were significantly lower than those in control eyes (*P* < 0.05). The VLD of the parafoveal average, the superior, inferior, nasal quadrants of the parafoveal region, and the nasal quadrant of the perifoveal region in fellow eyes were significantly lower than those in control eyes (*P* < 0.05) (Tables 5 and 6). Representative OCTA images are shown in Fig. 2.

**Table 5:** VLD of the subjects

VLD(mm^−1^)	Amblyopia eye *N* = 16	Contralateral eye *N* = 16	Control eye *N* = 19
	mean ± SD	mean ± SD	mean ± SD
Foveal	9.538 ± 4.176	10.756 ± 3.768	11.550 ± 2.960
Parafoveal average	21.694 ± 1.087	22.319 ± 0.697	22.756 ± 0.621
Superior	21.794 ± 1.470	22.588 ± 0.851	22.956 ± 0.837
Temporal	21.606 ± 1.301	21.919 ± 1.036	22.500 ± 1.114
Inferior	21.663 ± 1.439	22.400 ± 0.988	22.775 ± 0.864
Nasal	21.706 ± 1.558	22.331 ± 1.061	22.781 ± 0.935
Perifoveal average	18.480 ± 1.169	18.887 ± 0.877	19.000 ± 0.862
Superior	18.680 ± 0.947	19.113 ± 0.639	19.089 ± 0.798
Temporal	16.700 ± 2.999	17.547 ± 1.393	17.628 ± 1.733
Inferior	19.040 ± 1.101	18.900 ± 1.533	19.167 ± 0.842
Nasal	19.520 ± 0.718	19.927 ± 0.641	20.089 ± 0.419

*VLD* vessel length density, *SD* standard deviation

**Table 6:** Comparison of the VLD

VLD parameters	Amblyopia eye with contralateral eye	Contralateral eye with control eye	Amblyopia eye with control eye
	*P* value	*P* value	*P* value
Foveal	0.014*	0.154	0.126
Parafoveal average	0.110	0.010*	0.002*
Superior	0.111	0.023*	0.010*
Temporal	0.482	0.099	0.045*
Inferior	0.169	0.010*	0.013*
Nasal	0.204	0.027*	0.025*
Perifoveal average	0.259	0.125	0.152
Superior	0.131	0.097	0.188
Temporal	0.289	0.181	0.275
Inferior	0.776	0.988	0.711
Nasal	0.009*	0.026*	0.013*

*VLD* vessel length density

^*^P < 0.05, the comparison between the amblyopic eye group and the contralateral eye group used the paired sample t test, and the comparison between the amblyopic eye group, the contralateral eye group and the normal control group used the independent sample t test

### Perfusion density (PD)

**Fig. 3:**
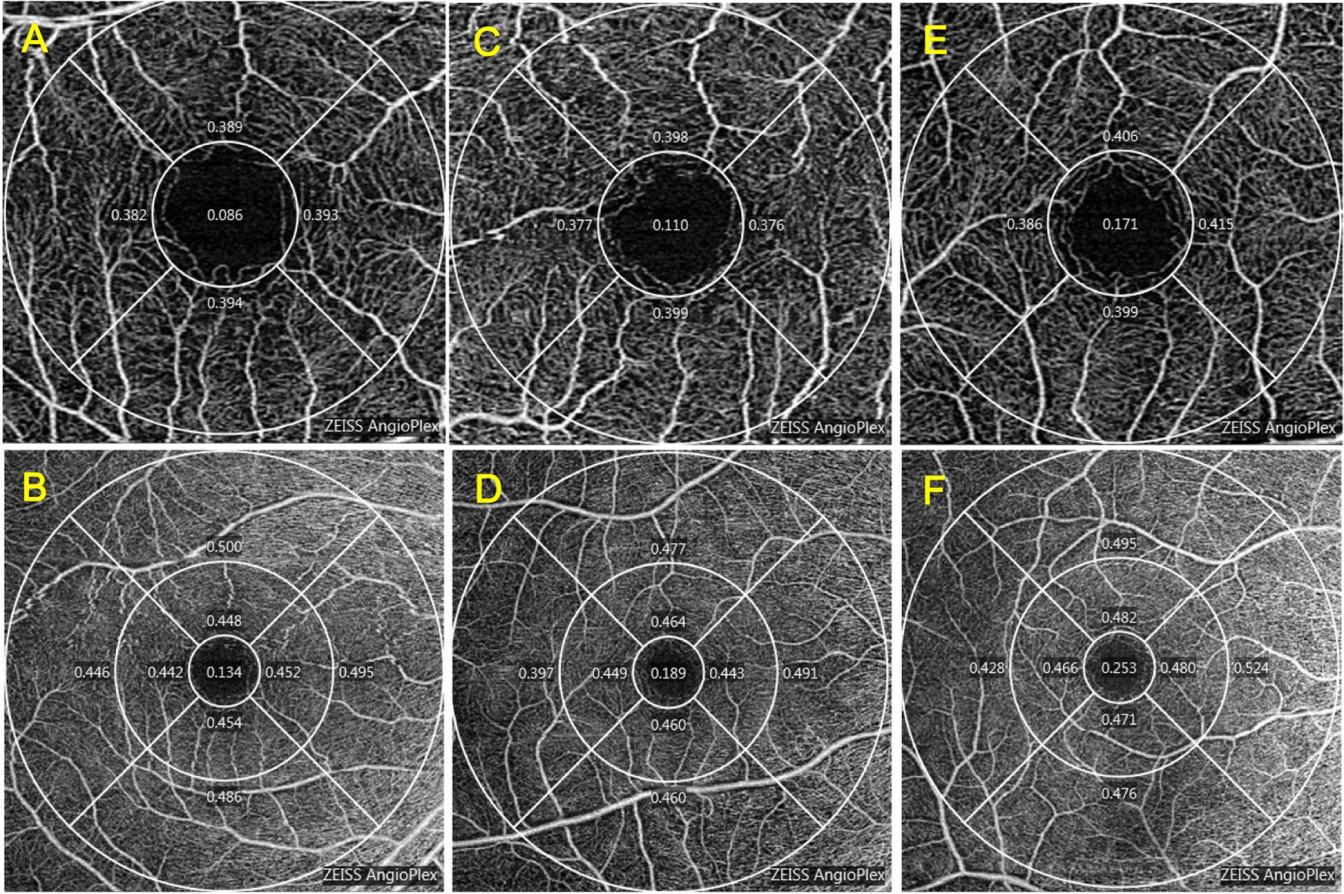
OCTA representative image of PD. **A** and **B** are amblyopic eyes, **C** and **D** are contralateral eyes, and **E** and **F** are control eyes. The scanning range of **A**, **C** and **E** was 3 mm × 3 mm in the macular area. The scanning range of **B**, **D** and **F** was 6 mm × 6 mm in the macular area. OCTA, optical coherence tomography angiography. PD, perfusion density

The PD of the foveal average in anisometropic amblyopic eyes was significantly lower than that in fellow eyes (*P* < 0.05). The PD of the parafoveal average and the inferior quadrant of the parafoveal region in anisometropic amblyopic eyes were significantly lower than those in control eyes (*P* < 0.05). The PD of the parafoveal average, and the inferior quadrant of the parafoveal region in fellow eyes were significantly lower than those in control eyes (*P* < 0.05) (Tables 7 and 8). Representative OCTA images are shown in Fig. 3.

**Table 7:** PD of the subjects

PD parameters	Amblyopia eye *N* = 16	Contralateral eye *N* = 16	Control eye *N* = 19
	mean ± SD	mean ± SD	mean ± SD
Foveal	0.169 ± 0.074	0.190 ± 0.069	0.202 ± 0.053
Parafoveal average	0.393 ± 0.021	0.400 ± 0.015	0.408 ± 0.014
Superior	0.398 ± 0.029	0.403 ± 0.018	0.414 ± 0.018
Temporal	0.396 ± 0.025	0.391 ± 0.019	0.404 ± 0.027
Inferior	0.386 ± 0.025	0.402 ± 0.026	0.407 ± 0.018
Nasal	0.391 ± 0.033	0.404 ± 0.022	0.408 ± 0.022
Perifoveal average	0.464 ± 0.030	0.471 ± 0.022	0.467 ± 0.029
Superior	0.473 ± 0.023	0.482 ± 0.016	0.480 ± 0.022
Temporal	0.413 ± 0.079	0.434 ± 0.041	0.434 ± 0.049
Inferior	0.483 ± 0.028	0.476 ± 0.040	0.484 ± 0.020
Nasal	0.486 ± 0.017	0.491 ± 0.017	0.494 ± 0.012

*PD* perfusion density, *SD* standard deviation

**Table 8:** Comparison of the PD

PD parameters	Amblyopia eye with contralateral eye	Contralateral eye with control eye	Amblyopia eye with control eye
	*P* value	*P* value	*P* value
Foveal	0.019*	0.188	0.157
Parafoveal average	0.298	0.035*	0.023*
Superior	0.598	0.206	0.077
Temporal	0.548	0.840	0.397
Inferior	0.089	0.014*	0.013*
Nasal	0.175	0.125	0.104
Perifoveal average	0.467	0.547	0.742
Superior	0.240	0.232	0.412
Temporal	0.330	0.247	0.358
Inferior	0.602	0.792	0.864
Nasal	0.203	0.183	0.154

*PD* perfusion density

^*^P < 0.05, the comparison between the amblyopic eye group and the contralateral eye group used the paired sample t test, and the comparison between the amblyopic eye group, the contralateral eye group and the normal control group used the independent sample t test

## Discussion

Most studies on the application of OCTA in children with anisometropic amblyopia and strabismus amblyopia found that there was no significant difference in FAZ area between amblyopic eyes and normal control eyes [8–10, 12, 13], which was consistent with our results. Our study found that the FAZ area of ​​the amblyopic eye group was significantly larger than that of the fellow eye group. There was no significant difference in FAZ perimeter and circularity in the amblyopic eye group and the fellow eye compared with the control eye. FAZ is the area without perfusion in the fovea. FAZ area, perimeter and circularity are quantitative indicators measured by OCTA through automatic identification, reflecting the structure of retinal blood vessels and blood flow density. Therefore, the results of this study suggested that the retinal vascular structure in children with amblyopia is roughly normal.

In previous studies on retinal perfusion, some studies found that the blood flow density of amblyopic eyes decreased [8, 9, 12–15]. Yilmaz and colleagues included 15 patients with strabismic amblyopia (mean age, 8.2 ± 2.3 years) and 15 age-matched controls (mean age, 8.6 ± 2.2 years). They reported that the mean superficial FAZ measurements did not differ from those of the control subjects. The vessel density of the superficial capillary plexus of eyes with amblyopia is lower than that of the fellow eye and age-matched controls [8]. Lonngi et al. included 59 children with 13 amblyopic eyes (mean age, 8.0 years) and 50 control eyes (mean age, 10.3 years). They found that the vessel density in the superficial capillary plexus in patients with amblyopia was lower than that in normal controls [9]. Others reported that the blood flow density of amblyopic eyes did not change obviously [10, 11]. Demirayak et al. involved 49 eyes from 17 patients with amblyopia (mean age, 8.6 ± 2.5 years) and 21 healthy children (mean age, 9.6 ± 2.9 years). In this study, macular vessel density (VD) (%) was calculated using ImageJ software. The VD (%) was calculated using the following formula: VD (%) = vascular area (pixels) / (region of interest – FAZ area) (pixels) × 100. They found no difference between amblyopic eyes, controls, and fellow eyes of patients with unilateral amblyopia in the vessel density of the superficial capillary plexus [10]. Our study is more consistent with the former. We showed that in children with anisometropic amblyopia, the blood flow density of the macula and the surrounding retinal capillary network was reduced. Compared with the fellow eye, the blood flow density of the amblyopic eye was reduced. According to the studies, the inconsistency of the research results may be caused by the difference in the measurement method. We measured perfusion parameters of the superficial retinal layer by built-in software. Demirayak et al. calculated macular VD (%) by using ImageJ software. Different measurement methods may lead to distinct research results.

Yilmaz et al. hypothesize that decreased vessel density in amblyopic eyes may be secondary to retinal or choroid microvasculature alterations, which are secondary to underuse [8]. Lonngi et al. speculated that the decrease in blood flow density in amblyopic eyes may be related to abnormal development due to lack of normal visual experience [9]. Cheung et al. found that the decrease in blood flow density was related to short axial length in healthy children. They believed that this may also be caused by amplification errors [16]. However, other studies have reported that there is no significant correlation between the axial length and refractive power of amblyopic eyes and the related parameters of retinal blood vessels [13]. In addition, we found that the vessel density of the fellow eyes also reduced compared with healthy controls. Therefore, we believe that there is a decrease in retinal vessel density in amblyopia children, and the vessel density in the fellow eye with normal refraction is also affected to some extent. However, due to few previous studies, the specific mechanism is still unclear. Therefore, further research is expected in the future.

Our study has presented the foveal, parafoveal and perifoveal regions of the VLD and PD of the superficial retinal layer and FAZ measurements of anisometropic amblyopic, fellow, and healthy children’s eyes obtained via OCTA for the first time.

The current study also has the following limitations. First, this study is a single-center study, and only one measurement was performed. Dynamic changes in various parameters of retinal blood flow density in children with anisometropic amblyopia were not observed. In the future, large samples and longitudinal studies are still needed to further confirm that retinal blood flow density parameters can be used and whether they are an effective indicator for follow-up monitoring of the visual prognosis of children with anisometropic amblyopia. Second, limited by the current OCTA technology, considering the impact of projection artifacts on the accuracy of the quantitative analysis of the blood flow density of the deep retina and choroidal capillaries, this study only performed blood in the 3 mm × 3 mm and 6 mm × 6 mm areas of the superficial retina in the macular area. It is expected that wide-angle OCTA technology that is less affected by projection artifacts will be used in the future to conduct more in-depth research on the deep blood vessels and choroid of the whole retina in children with amblyopia.

## Conclusions

In children with anisometropic amblyopia, the blood flow density of the macula and the surrounding retinal capillary network were reduced. This study provides a certain clinical basis for the future study of the pathogenesis of amblyopia and the application of OCTA technology for the diagnosis and follow-up of children with amblyopia.

## Data Availability

The data that support the findings of this study are available from the corresponding author on reasonable request.

## Abbreviations

AL: Axial Length
BCVA: Best Corrected Visual Acuity
FAZ: Foveal Avascular Zone
IOP: Intraocular Pressure
LogMAR: Logarithm of the Minimum Angle of Resolution
OCTA: Optical Coherence Tomography Angiography
PD: Perfusion Density
SD: Standard Deviation
VD: Vessel Density
VLD: Vessel Length Density
